# Sex-dependent performance differences in curvilinear aiming arm movements in octogenarians

**DOI:** 10.1038/s41598-023-36889-5

**Published:** 2023-06-16

**Authors:** Dieter F. Kutz, Stephanie Fröhlich, Julian Rudisch, Katrin Müller, Claudia Voelcker-Rehage

**Affiliations:** 1 Department of Neuromotor Behavior and Exercise, Institute of Sport and Exercise Sciences, University of Muenster, Wilhelm-Schickard-Str. 8, 48149 Münster, Germany; 2 Faculty of Behavioural and Social Sciences, Institute of Human Movement Science and Health, Chemnitz University of Technology, 09107 Chemnitz, Germany

**Keywords:** Neurological disorders, Cognitive ageing, Predictive markers

## Abstract

In an aging society, it is necessary to detect the cognitive decline of individuals at an early stage using simple measurement methods. This makes early health care possible for those affected. The aim of the study was to develop a classifier for cognitive state in older adults with and without mild cognitive impairment (MCI) based on kinematic parameters of linear and curvilinear aiming arm movements. In a group of 224 older adults over 80 years of age (cognitively healthy and MCI), the movement duration and intersegment intervals of linear and curvilinear arm movements of 20 cm were recorded. Movement duration was significantly longer in the curvilinear condition than in the straight movement, and MCI participants required significantly more time than cognitively healthy participants. Post-hoc analysis on the fluidity of movement in the curvilinear condition showed that MCI men had significantly longer inter-segmental intervals than non-MCI men. No difference was found in women. Based on the inter-segmental intervals, a simple classifier could be developed that correctly classified 63% of the men. In summary, aiming arm movements are only conditionally suitable as a classifier for cognitive states. For the construction of an ideal classifier, age-related degeneration of cortical and subcortical motor areas should be considered.

## Introduction

Aging is a progressive biological process that leads to structural and functional changes that reduce one’s ability to adapt to environmental stressors^1,2^. Aging negatively affects cognition, so that even in physically healthy older adults, a slight decline in brain function is observed, which predisposes them to dementia later in life^3–5^. Specifically, fluid intelligence (e.g., perceptual speed, memory, reasoning, verbal knowledge, and verbal fluency) has been shown to decline continuously over the course of life after age 20^3,5^. The decline increases between ages 60 and 70^3,5^ and correlates with hippocampal volume decline^3^. Large studies have shown that the incidence of dementia increases rapidly after age 65, doubling every 5 years^6^, with Alzheimer’s disease (AD) accounting for 60% to 70% of all cases^7^. In Germany, according to the German Alzheimer Society^8^, the number of dementia patients will increase to three million by 2050. Compared to the prevalence of dementia, the prevalence of a mild cognitive impairment (MCI) is more than double^9^. Further, in AD the progression of MCI to dementia is estimated to occur in 10% of affected individuals per year^9,10^. The public health implications are enormous, because AD is also associated with an increased risk of death in addition to incident dementia^11^. Early detection of dementia and its precursors can enable more sustainable disease management and optimal health care for those affected^12^. Detected early enough, people with a precursor of dementia (e.g., MCI) could begin programs that help them maintain or change their lifestyles. Many psychometric questionnaires are based on subjective patient reports. These reports may be inaccurate due to disease-related memory impairment and associated loss of insight into the progression of one’s own disease^13,14^. Alternatively, reports from physicians or family members and caregivers can be used^13–15^. For the latter two groups, prior instruction in the administration of the questionnaires is necessary^13^. Therefore, the validity of patient questionnaires is limited. Yet, difficulties exist in accurately identifying MCI and early stages of dementia in the population at large using traditional psychometric testing, so it is desirable to develop alternative or additional methods (e.g., easily administered motor tests) for case ascertainment.

Older adults show decreased motor performance, which has been attributed to three factors^16^: a decrease in central and peripheral sensorimotor functions (e.g., increased noise, reduced tactile sensation), a reduction of information processing (e.g., slowing of movement), and/or changes in the motor system (e.g., loss of motor units, motor unit reorganization) that lead, e.g., to a decreased ability to produce a consistent force result. As such, older subjects show an increased proportion of secondary partial movements in rapid aiming tasks compared to younger subjects^16–18^. According to the optimized submovement model^19^, motor planning produces a primarily ballistic movement, whereas sensory feedback guides secondary corrective movements during movement execution. Pauses (inter-segment intervals, or ISIs) sometimes occur between each segment of the movement, and these pauses are generally longer or more variable when participants’ motor control or motor planning is less developed or reduced (e.g.^17,18,20,21^). Therefore, measuring ISIs during aiming movements is a useful measure for assessing human arm movement control^18,21^. In this context, good motor programming during fast and multi-segment aiming movements is characterized by short movement durations and short ISIs, whereas reduced motor planning ability (such as in MCI or dementia) is characterized by prolonged movement durations and/or ISIs^22–25^. This is especially observed in diadochokinetic movements, such as finger tapping (e.g.^26^).

The aim of this study was to investigate the effects of cognitive state on movement planning and movement production in people over 80 years old. To this end, participants performed two fast aiming arm movements (linear condition and curvilinear condition) with a pen on a tablet. Outcome measures were movement duration and ISIs, with MCI participants expected to perform the tasks more slowly and haltingly than adults without MCI. To classify MCI, participants completed a neuropsychological test battery that included the German version of the MoCA^27^ and the German version of the Consortium to Establish a Registry for Alzheimer’s Disease Neuropsychological Test Battery (CERAD-NP)^28^. The CERAD-NP also includes the Mini-Mental State Examination (MMSE). The MoCA was used to measure global cognitive performance and screen for MCI and has a higher sensitivity for mild cognitive impairment than the MMSE^29^. In addition, we aimed to investigate whether the group differences found were suitable to serve as classifiers for the two cognitive groups. Finger tapping experiments with self-selected or fast paced tapping behavior were also conducted with the same sample of subjects^26^. The results of the finger tapping experiments (e.g. tap-cycle, tap-duration, force-peak, velocities of flexion and extension as well as the duration of application of constant force to the force plate and the duration without contact to the force plate) were also used as a classifier for the cognitive groups^26^. Therefore, the classification result of the aiming arm movements was compared with the result of the finger tapping experiments. Since the investigation of the tapping parameters revealed a sex-dependent difference in some parameters^26^, we further investigated whether women and men solve the two aiming tasks differently. The investigation of the movement results will lead to a better understanding of the basis of motor planning in older adults.

## Results

This study investigated the performance of 224 participants over 80 years old while making aiming arm movements consisting of drawing linear and curvilinear lines. Demographic characteristics as well as measures for fine motor skills are reported in Table 1. In particular, fluency by means of ISIs were examined; that is, we examined whether the movements were performed in one motion or haltingly. To minimize the influence of corrective movements to the beginning or end of the movement, the analysis was limited to the middle half of the distance (i.e., 10 cm).

**Table 1:** Characteristics of the groups according to sex and cognitive status.

	Female (*n* = 108)		Male (*n* = 116)	
	Non-MCI (*n* = 81)	MCI (*n* = 27)	Non-MCI (*n* = 78)	MCI (*n* = 38)
Age in years	82.1 ± 0.3	82.8 ± 0.5	82.5 ± 0.2	82.8 ± 0.5
Education in years^a^	13.0 ± 0.3	12.2 ± 0.4	15.3 ± 0.4	14.9 ± 0.5
MMSE (0–30)	28.2 ± 0.2^b^	26.8 ± 0.4	27.9 ± 0.2	27.6 ± 0.3
MoCA (0–30)^a^	27.2 ± 0.2^b^	22.9 ± 0.3	26.2 ± 0.2^b^	22.6 ± 0.3
FM score (1–6)	5.0 ± 0.1	5.0 ± 0.2	5.2 ± 0.1	5.5 ± 0.1
CERAD drawing score (0–11)	10.6 ± 0.1	10.3 ± 0.2	10.7 ± 0.1	10.6 ± 0.1
CERAD TMT A [s]	56.7 ± 2.6	63.6 ± 3.2	58.0 ± 2.4	62.4 ± .2^c^

Given are means ± SEM.

*non-MCI* cognitively healthy individuals, *MCI* mildly cognitively impaired individuals. For details of the classification, see text. *f* female, *m* male, *MMSE* Mini-Mental State Examination, *MoCA* Montreal Cognitive Assessment, *FM score* fine motor score, see text. CERAD drawing score: sum of the scores for drawing a circle, rhombus, rectangle, and cube. *CERAD TMT A* Duration of the Trial Marking Test A.

^a^Significant difference between female and male participants (Kruskal–Wallis test, *P* < 0.05).

^b^Significant difference between non-MCI and MCI within one sex (Kruskal–Wallis test, *P* < 0.05).

^c^Significant difference between non-MCI and MCI participants independent of sex (Kruskal–Wallis test, *P* < 0.05).

The analysis of the movement duration showed a significant main effect for the factor condition, with longer durations in the curvilinear than in the linear condition (medians: 699 ms vs 1215 ms, respectively; Kruskal–Wallis test, χ^2^(1) = 113.18, *P* < 0.001, effect size φ = 0.72). MCI participants needed more time to perform the movements than non-MCI participants (medians: 1073 ms vs 925 ms, respectively; Kruskal–Wallis test, χ^2^(1) = 6.84, *P* < 0.009, φ = 0.18). A sex-dependent difference in the duration of all movements could not be shown (median(female): 961 ms, median(male): 971 ms; Kruskal–Wallis test, χ^2^(1) = 0.001, *P* < 0.970).

The analysis of movement durations by condition showed that for the condition linear, a main effect was found for the factor cognitive state on trend level (Kruskal–Wallis test, χ^2^(1) = 3.328, *P* = 0.068, φ = 0.12) but no significant effect was found for the factor sex (Kruskal–Wallis test, χ^2^(1) = 0.220, *P* = 0.639) nor for the interaction of cognitive state and sex (Kruskal–Wallis test, χ^2^(3) = 3.726, *P* = 0.293). For the curvilinear condition, a significant effect was found for the factor cognitive state (Kruskal–Wallis test, χ^2^(1) = 6.358, *P* = 0.012, , φ = 0.17), but no effect was found for sex (Kruskal–Wallis test, χ^2^(1) = 0.661, *P* = 0.416) and an effect on the trend level was found for the interaction cognitive state and sex (Kruskal–Wallis test, χ^2^(3) = 7.046, *P* = 0.070, φ_c_ = 0.13). The movement durations divided by condition, cognitive state, and sex are reported in Table 2.

**Table 2:** Movement duration for the linear and curvilinear condition of female and male participants in ms.

Condition		Female		Male	
		Non-MCI (81)	MCI (27)	Non-MCI (78)	MCI (38)
Linear	Median/IQR	662/558	792/733	674/480	708/452
	Range	[223, 3177]	[226, 3538]	[127, 2635]	[275, 3200]
Curvilinear	Median/IQR	1182/636	1232/653	1122/484	1378/625
	Range	[491, 4052]	[685, 2841]	[416, 2914]	[667, 3785]

Data are separated by their cognitive state as healthy participants (non-MCI) and mildly cognitively impaired individuals (MCI). The number of participants in each group is given in parentheses. Indicated are the median, interquartile range (IQR), and data range.

### Inter-segment intervals (ISIs)

**Figure 1:**
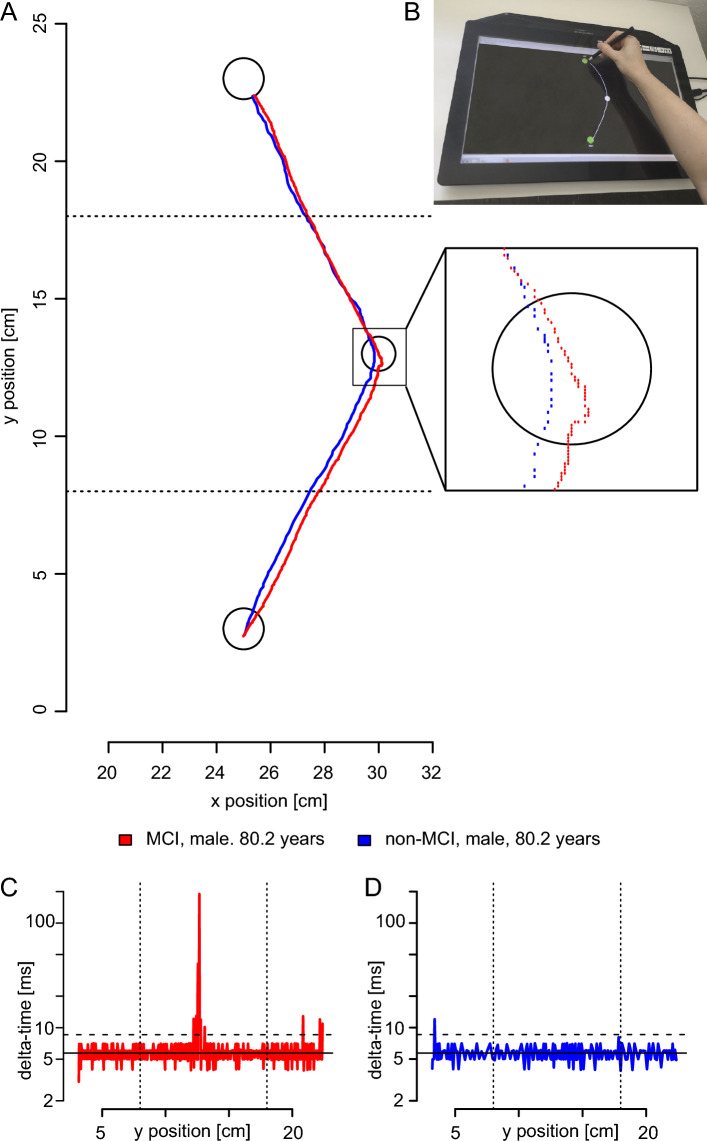
Methods. (**A**) Characteristic examples of drawing motion of a non-MCI participant (blue line) and an MCI participant (red line). Only the drawing movement between the horizontal dashed lines was used in the analysis, i.e., in the range from 8 to 18 cm for the vertical position of the tablet. The individual sequence of measuring points during the drawing movement in the area of the middle circle is shown in the inset; x-axis = x position and y-axis = y position of the tablet in cm. (**B**) The experimental setup. (**C,D**) Time difference between the individual measurement points of the drawing movement for an MCI participant (**C**) and for a non-MCI participant (**D**). The vertical dashed lines in (**C,D**) indicate the range of the y position used for further analysis (see **A**). The horizontal thin line indicates the mean sampling interval of about 5.7 ms, and the dashed horizontal line indicates the threshold of 1.5 times the sampling interval (8.6 ms).

**Figure 2:**
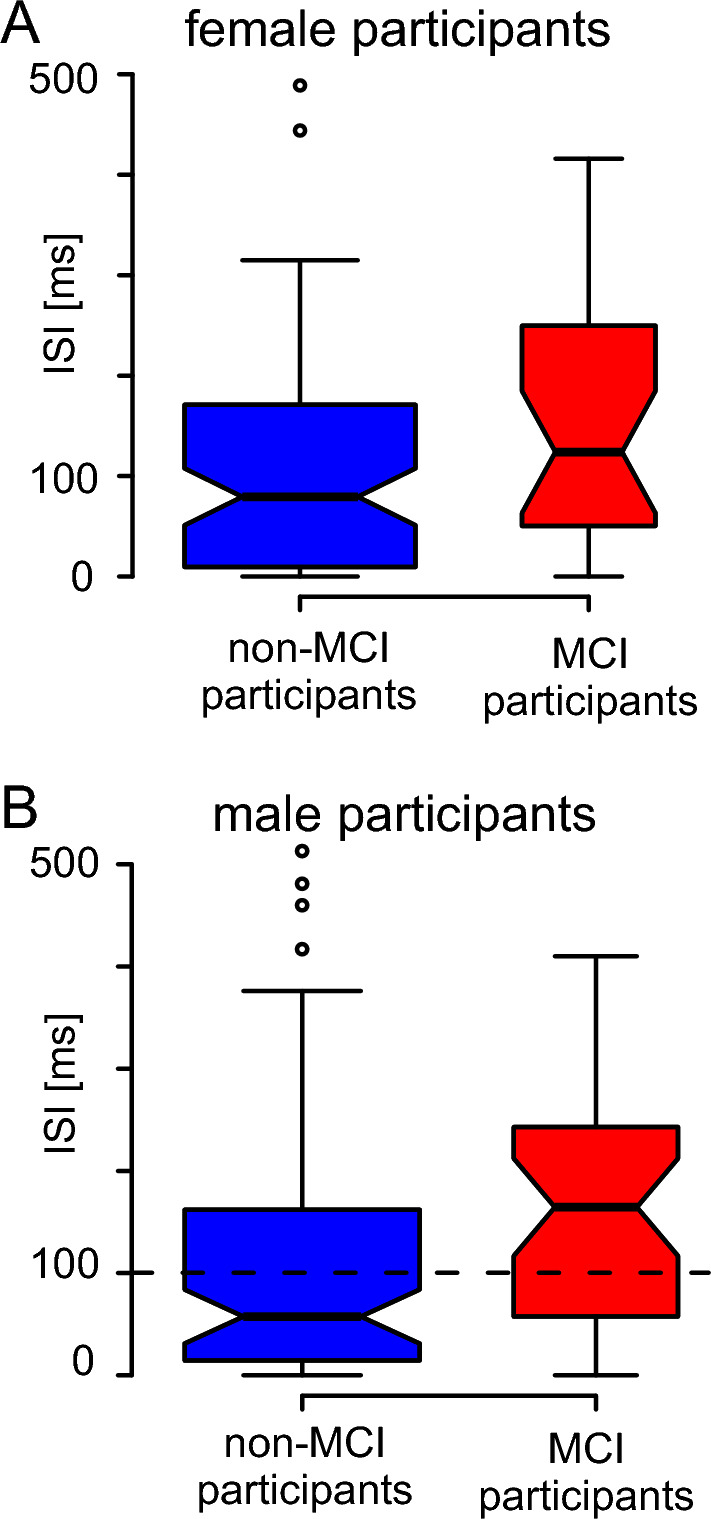
Boxplots of inter-segment intervals (ISIs) in the curvilinear condition. (**A**) Female participants, (**B**) male participants. The boxes represent the mean 50th percentile, the thick line represents the median, and the whiskers the range of data belonging to each group. Outliers are represented as circles. Outliers above 500 ms are not displayed for visual reasons. Blue boxplots indicate the distribution of non-MCI participants and red boxplots of MCI participants. The width of each boxplot reflects the relative proportion of the number of participants. The notches reflect the estimator of the 95% confidence interval of the median. The dashed line in B indicates the classification threshold of 100.4 ms.

In the curvilinear condition, halting movements occurred in the region of the middle point (Fig. 1A). At this point, it was necessary to convert the abduction in the shoulder and combined extension and pronation in the elbow performed at the beginning of the movement into adduction in the shoulder and combined flexion and supination in the elbow. Thus, diadochokinesis was necessary to execute the movements of both segments. MCI participants showed halting movements with ISIs at the time of movement change. A characteristic example for an MCI participant (Fig. 1A inset, red points) showed a dense accumulation of points and abrupt, choppy changes in direction of movement. These uneven movements with halting were also evident in the time differences of the data points, which could be 100 ms or more (Fig. 1C, delta-time at ~ 12.5 cm y position). In contrast, the example for a non-MCI participant (Fig. 1A inset, blue points) showed a fairly even spacing between the individual data points, indicating that the movement was performed as one segment with the same average speed. The time intervals of the individual data points in this area corresponded to the sample interval (Fig. 1D), and there was no halting of the movement. The halting movements occurred more frequently and for a longer time in MCI men than in all other groups (Fig. 2). The analysis of the ISIs showed a significant main effect for the factor cognitive state with longer duration for MCI participants (medians: 145 ms vs 94 ms, respectively; Kruskal–Wallis test, χ^2^(1) = 9.682, *P* = 0.002, φ = 0.21), no difference for the factor sex (female: 82 ms, male: 84 ms; Kruskal–Wallis test, χ^2^(1) = 0.541, *P* = 0.462) and a significant effect for the interaction of cognitive state and sex (Kruskal–Wallis test, χ^2^(3) = 10.511, *P* = 0.015, φ_c_ = 0.15). The ISIs divided by cognitive state and sex are shown in Fig. 2. The post-hoc analysis on the fluidity of movement in the curvilinear condition showed that MCI men had significantly longer ISIs than non-MCI men, whereas no difference was found in women (male: *P* = 0.039, female: *P* = 0.972, both Bonferroni corrected; Fig. 2). Using the estimators of the 95% confidence interval of the medians (notches of the boxplots in Fig. 2B), a classification threshold of 100.4 ms was calculated for the male participants as the mean of the upper estimator of the non-MCI participants and the lower estimator of the MCI participants. Overall, 73/116 (63%, φ = 0.26, Table 3: curvilinear task) of the participants could be correctly classified, which was better than the theoretical value of 56% (see “Methods” section).

**Table 3:** Confusion matrix for male participants.

		Tapping task (prediction)		Curvilinear task (prediction)	
		Non-MCI	MCI	Non-MCI	MCI
(A) Based on tapping behavior and curvilinear aiming arm movement task					
Neuropsychological tests (real)	Non-MCI	**70**	8	**48**	30
	MCI	23	**15**	13	**25**
Sums		93	23	61	55

		Combining both tasks (prediction**)**			
		Non-MCI	MCI		
(B) Combining both tasks (see text)					
Neuropsychological tests (real)	Non-MCI	**44**	4		
	MCI	10	**12**		
Sums		54	16		

Correctly classified participants are given in bold font.

A comparison of the cognitive scales MoCA and MMSE as well as the years of education divided by cognitive status and sex shows that women had significant fewer years of education (Kruskal–Wallis test, χ^2^(1) = 27.647, *P* < 0.001, φ = 0.35), and higher MoCA score (Kruskal–Wallis test, χ^2^(1) = 11.523, *P* < 0.001, φ = 0.23) than men (Table 1). It further evidences that for the MMSE score, there is a significant difference between non-MCI and MCI females (Kruskal–Wallis test, χ^2^(1) = 8.387, *P* < 0.001, φ = 0.19) and for the MoCA score, there is a significant difference between non-MCI and MCI participants in both females and males (Kruskal–Wallis test, χ^2^(3) = 114.72, *P* < 0.001, φ_c_ = 0.41) (Table 1). Examination of participants’ the fine motor skills (see “Methods” section) revealed a significant difference between non-MCI and MCI participants only for the CERAD TMT A score independent of the sexes (Kruskal–Wallis test, χ^2^(1) = 4.6286, *P* = 0.031, φ = 0.14). Regression analysis between ISIs and the TMT A score show that the differences are given by the MCI men (slope = 2.76, *P* = 0.006, F(1, 36) = 9.993, adjusted R^2^ = 0.20).

### Comparison of tapping and aiming arm behavior

In a recently published study^26^, we reported the possibility of classifying participants based on selected movement parameters of a tapping task during self-selected or fast pace. For the 116 male participants from this current study, the tapping parameters were additionally analyzed and the participants were classified accordingly. Classification using tapping algorithm classified 93 participants as non-MCI (of which 70 were correctly classified) and 23 participants as MCI (of which 15 were correctly classified, φ = 0.32, Table 3A), giving it a sensitivity of 39% and a specificity of 90%. When the aiming behavior of the same 116 male participants in the current study was examined, 61 participants were classified as non-MCI (of which 48 were correctly classified) and 55 participants as MCI (of which 25 were correctly classified φ = 0.29, Table 3A). In summary, the sensitivity was 66% and the specificity 62%. Combining both algorithms in the form of a logical AND, that is, those classified as non-MCI in the tapping algorithm must additionally have ISIs < 100.4 ms and those classified as MCI must also have ISIs ≥ 100.4 ms could be performed on a subset of 70 participants. This resulted in 54 participants classified as non-MCI and 16 classified as MCI. The total sensitivity was 12/22 (55%) and the specificity 44/48 (92%) with an effect size of φ = 0.48 (Table 3B: combining both tasks). Thus, it appears that the combination of the two examinations improved the classification of the male participants.

## Discussion

This study examined the performance of participants over 80 years old when making linear and curvilinear aiming arm movements. Results showed that some of the differences between female and male participants could be attributed to a change in behavior of MCI males. They performed the curvilinear movement more haltingly and with longer ISIs. Based on this time difference, a simple classifier could be developed that correctly classified 63% of the men. Combining this classification with the classification based on tapping parameters^26^, such that participants were classified as either non-MCI or MCI in both algorithms, showed that of the men so classified, 44 of the 54 (81%) classified as non-MCI men and 12 of the 16 (75%) classified as MCI men were correctly identified. On the other hand, it should be noted that no acceptable identification was found for 46 out of 116 men performing both tests.

Our study design was guided by the study of Yan^21^, who measured linear and curvilinear arm movements in 20 participants over 80 years old. For a total distance of 20 cm, his participants needed ~ 700 ms for the linear and about 1330 ms for the curvilinear condition (Fig. 5 in Ref.^21^). This was significantly faster than what was found in this study. It should be noted that in Yan’s study^21^, the older participants had 3–8 practice trials and then repeated the task until 10 successful trials per condition were measured. They also had to respond to a start signal in a range of 150 to 500 ms. In our study, the subjects had no practice trials, and 7 trials without repetition were measured. In addition, participants started each trial independently. It can therefore be assumed that the participants in our study performed the task with more calmness and caution. Another difference is the index of difficulty (ID) according to Fitt’s law^30^. With higher ID, the movement duration increases and the ISI increases, especially in older subjects^31^. In the linear condition, the ID in Yan’s study was 4.3^21^, whereas in this study it was 4.7. For the curvilinear condition, the ID for the movement to the middle circle in Yan’s study was 2.5 and for the subsequent movement to the target 3.5^21^. In our study, the corresponding values were 4.2 and 3.9. Hence, movements were more difficult in our study, and this might explain the temporal differences.

Few studies have investigated hand movements in the prodromal stage of dementia such as MCI (for review, see^23^). Kinematic parameters such as outcome measure in aiming movements have only been used in two studies to discriminate healthy older participants from MCI participants. Comarda et al.^22^ showed in a comparable task for the linear condition that MCI patients (N = 11) took significantly longer to perform the movement than non-MCI participants (N = 11). Yan et al.^25^ came to the same conclusion when they studied a group of 10 non-MCI participants and 9 MCI participants. Thus, the results of our studies confirmed the previous exploratory studies on smaller groups. Furthermore, our experiments showed that there was a sex difference in the performance of the curvilinear condition. A significant difference was found for male participants in the total duration of ISIs between non-MCI and MCI participants. This difference allowed for separation of the two groups. A comparable difference was not found for female participants.

Our previous study on tapping behavior had shown that the force used for tapping was a relevant factor for distinguishing between groups as well as sexes^26^. It is therefore possible that the overall longer ISIs of the MCI men were caused by an excessive grip force and higher contact pressure of the pen on the tablet. Hertzum and Hornbaek^32^ showed that the movement parameters in aiming tasks differ when using a tablet and pen or an optical mouse, as they found that the use of a mouse required less force to move or hold. They also showed that movements with a pen were performed significantly slower than with a mouse, whereby this occurred more in older subjects^32^. Force measuring films have been shown to be suitable for measuring the grip forces and position of individual fingers^33,34^ as well as their dynamics during movement^35^. Findings from writing studies have shown that applying these films on a pen is possible^36,37^. Therefore, it is recommended that such systems are used in the future to further elucidate the forces during the drawing behavior of MCI men. That MCI males show a different drawing behavior is also evident from the TMT A scores. Only for this subgroup a correlation between ISIs and the TMT A score could be shown. Therefore, it can be assumed that the measurement of ISIs in drawing movements with multiple directional changes, as they occur in the TMT A, allows a better discrimination of MCI males. That there are no other correlations with the other two fine motor skill measures is due to the respective assessment of these measures. For example, the CERAD drawing score assesses the quality of a drawing and not its execution. The FM score is a self-reported frequency of performing at least one of a selection of fine motor skills (playing a musical instrument, typing on a keyboard, writing, needlework, model building or other fine motor skills). The high values of the FM score are noteworthy (Table 1). This is an indication that there is a selection bias in the recruitment of participants. Thus, an explicit inclusion criterion was that the participants manage the way to and from the laboratory by means of self-organized transport. Thus, they are not representative of all people over 80 years of age. On the other hand, the entire study protocol was explicitly developed for this group^12^.

A limitation of this study is the discontinuous data collection of the tablet. This resulted in an uneven and unpredictable loss of data points over time. Therefore, higher mathematical techniques such as jerk analysis were not useful. Nevertheless, a simple technique for evaluating target movements can be derived from this study. Effectively, the non-transmission of the data acts as a high-pass filter of velocity, since a continuous transmission of the pen position only occurred for a minimum movement speed of the pen. It is technically possible to set up a filter even with continuous data acquisition. By doing so, the duration of falling below the velocity threshold can simply be measured, and the sum of the ISIs can be read.

In addition to the experimental conditions, the neurological status of the participants must also be considered. All participants reported no neurological deficits (an exclusion criterion; see “Methods” section). However, individuals may have had varying degrees of age-related degeneration and in different relevant areas of the CNS (e.g., cortex, spinal cord, basal ganglia, cerebellum). It is known that the cerebellum is generally important for the coordination of motor performance, such as diadochokinesis. Limitations in aiming behavior can be explained not only by cognitive impairment but also by age-related cerebellar decline^38–41^. Different portions of the cerebellum correlate with motor abilities than correlate with cognitive abilities (reviewed in Ref.^26^). Age-related degeneration of the motor cerebellum is comparable to degeneration in cerebellar diseases^39^. In the aiming task, it can be assumed that the degeneration of the motor cerebellum has an influence on performance. Therefore, the lack of definite classification in 46 of 116 men may be explained by varying degrees of cerebellar degeneration. Overall, the two tasks differ in their use of arm/hand muscles, with a greater emphasis on finger muscles in tapping. Considering the different innervation of cortical and sub-cortical areas of the arms and fingers^42^, the individual variations in task performance may indicate different impairments of brain areas.

Overall, the possible degeneration of relevant motor systems (e.g., the cerebellum) should be considered when examining the cognitive state by means of movements. First, additional motor tests can be performed to specifically determine the degeneration of the above mentioned areas^26^. Second, different tasks can be combined whose results allow the construction of a classifier (e.g., tapping behavior, aiming arm behavior) to increase sensitivity or specificity.

## Methods

This study is part of the SENDA study (Sensor-based systems for early detection of dementia, registered in the German Clinical Trials Register under DRKS00013167), which was conducted at Chemnitz University of Technology, Germany. The detailed study protocol was published earlier^12^. Sample size calculation showed that 200 participants are required for analysis with α = 0.05 and power = 0.80^12^. Only information relevant to the current research question is described here.

### Participants

The SENDA study was advertised by local general practitioners and in newspapers. In total, 244 participants (123 males; age 79–93 years; M = 82.5; SD = 2.5) took part in the study and were recruited from January 2018 to March 2020. Study participation required walking ability, sufficient German language skills, residence in or around Chemnitz, Germany, and a self-organized means of travel to and from the laboratory. Volunteers were excluded before testing if any of the following criteria applied: (1) acute psychological disorder; (2) diagnosis of any neurocognitive or neurological disorder; (3) past traumatic head injury; (4) substance abuse; (5) participation in other clinical studies; (6) a physician-directed ban from physical activities; (7) severe restrictions due to cardiovascular, pulmonary, or orthopedic diseases; or (8) failure to reach the minimum required score of 19 during screening with the Montreal Cognitive Assessment (MoCA)^27^. Each participant received a total of 25 EUR compensation for each measurement time point with three individual appointments for their participation.

The analysis for this article included 224 participants who took part at the baseline measurement. Exclusion from analysis was due to dropout from the study before all needed testing was completed (n = 18) or technical issues during the recording (n = 2). Due to the participants’ old age, many followed a medication regimen (n = 200), which most often included medication for high blood pressure, thrombosis prophylaxis, cholesterol reduction, stomach acid reduction, or thyroid function.

### Neuropsychological testing and MCI classification

The neuropsychological testing and MCI classification are described in detail elsewhere^43^. Briefly, all participants went through an intensive neuropsychological test battery, which was carried out by trained testing staff at the university lab. The tests included the German version of the MoCA^27^ and the German version of the Consortium to Establish a Registry for Alzheimer’s Disease Neuropsychological Test Battery (CERAD-NP)^28^. The MoCA was used to measure global cognitive functioning and to screen for MCI. The MoCA is the second-most-utilized geriatric cognitive screening tool after the Mini-Mental State Examination (MMSE) and has superior sensitivity to mild cognitive impairments^29^. The CERAD-NP examines the cognitive domains of memory, language, executive functions, and visuo-construction. MCI classification was based on the recommendations of The National Institute on Aging and the Alzheimer’s Association^44^ and in accordance with the criteria proposed by^45^. Cognitive impairments were determined according to performance in MoCA (one sum score) and CERAD-NP (nine separate test scores). The following CERAD-NP scores were used: verbal fluency, Boston naming test, phonematic, constructional praxis, word list learning, word list recall, word list recognition, constructional praxis recall, and trail making test. We followed a two-step procedure recommended for diagnosis of MCI in the general population, which states that, first, a screening should be used, and, second, in the case of abnormal findings, in-depth cognitive testing should follow^46^. A MoCA score below 26 points and at least one CERAD-NP test performance at least 1.5 standard deviations below the normative mean (taking into consideration age, sex, and education level) resulted in classifying the participant as having mild cognitive impairments (MCI). All other participants were considered non-MCI according to Ref.^46^, because these individuals did not have abnormal values in screening (MoCA > 25) or show cognitive impairment in the in-depth clinical tests.

### Tasks and recording

Participants carried out three fine motor tasks^12^, including (1) force modulation of a precision grip with the thumb and index finger^47^; (2) tapping with the index finger of the right hand^26^; and (3) aiming arm movements by connecting dots on a touchscreen with a pen as studied by Yann^21^. Here we report the results of the third motor task, experiment (3) and compared them to the results of experiment (2). In experiment (2) participants tapped with one’s dominant index finger on the force transducer in two different conditions: at a self-selected pace or as fast as possible (see^26^). Task and experimental setup of experiment (3) have been reported elsewhere^12^ and are briefly summarized here. A touch monitor (Manufacturer: Hannstar Display Corp., 23.0 in., hardware resolution 1920 × 1080 pixels, Taipei City, Taiwan; Modell: HSG 1353) and a touch pen (WACOM Bamboo-Stylus Alpha CS-180, length 130 mm, diameter 9 mm, weight 12 g) were used. The monitor was placed horizontally on a table in front of the participant. The pen was held in the participant’s dominant hand (see Fig. 1B). The data acquisition and visual feedback during the experimental procedures in real time were programmed using a customized script for LabView 2015 (National Instruments, Austin, TA, USA). The program recorded the x and y positions of the pen and the time stamp of the measurement at an average sampling frequency of 175 Hz. Participants had to connect points on the touchscreen (black desktop background) with the pen by drawing a line. There were two tasks: drawing a linear and a curvilinear line. For the linear condition, two green dots (diameter 15 mm) were displayed on the screen aligned vertically, one marked ’Start’ and one ‘Target’ (see Fig. 1B). In the curvilinear condition, a third white intermediate point (diameter 12.5 mm) was presented halfway between the ‘Start’ and ‘Target’ dots (horizontal distance 25% of the ‘Start’–‘Target’ distance to the right), through which participants had to draw a curved line in one stroke (see Fig. 1B). Participants received feedback by having a white line displayed on the tablet’s screen. In both conditions (linear and curvilinear), two different distances (50 mm and 200 mm) between the ‘Start’ and ‘Target’ points were tested. In total, four conditions (linear short, linear long, curvilinear short, and curvilinear long) were performed as a block with seven trials in each condition, and the conditions appeared in a random order. Overall time to perform all four blocks was less than 8 min.

### Data analysis

The data analysis included two measurement variables: the movement duration and the inter-segment intervals (ISIs) for the fluidity of the individual movement. Each trial was started independently by the participants by placing the pen on the tablet. Some participants simultaneously placed the pen and their hand on the tablet when starting the trial; therefore, it was not always possible to clearly determine the start of the movement. While performing the task, the participant received visual feedback in the form of the drawn line, shown in white. The correct execution of the task with only the pen touching the tablet is shown in Fig. 1B. At the moment the pen touched the circular line of the circle ‘Target’, both the white line and the ‘Start’ and ‘Target’ circles disappeared. Therefore, visuomotor control at the end of the movement was different from that during the movement, and analysis of the movements was not comparable. To ensure that the movement analysis was not influenced by effects at ‘Start’ or around ‘Target’, a safety distance of 15 mm from the center of the ‘Start’/‘Target’ circles was excluded for analysis. As such, of the original 50 mm distance for the short conditions (linear short, curvilinear short), only a distance of 20 mm was available for analysis. This was too short, and these conditions were excluded from further analysis. For the 200 mm conditions, to exclude effects of the ‘Target’ and ‘Start’ areas on the movement, only the middle half of the movement (8–18 cm) for the long conditions was used for analysis. This area contained the position of the laterally offset circle in the center of the curvilinear condition. The movement duration resulted from the difference of the time stamps between the first measuring point above the 8-cm y position and the last measuring point below the 18-cm y position. The total duration of inter-segmental intervals (ISIs) was used as a measure of the fluidity of the movement. The detailed calculation of this measure is described in the following paragraph and was developed to fit the data-recording properties of the tablet.

The tablet measured a pen position only if the position changed by more than one pixel (0.265 mm edge length) within the average sampling interval of ~ 5.7 ms. This meant that if the pen was not moved by at least 1 pixel within a scan interval, no new data was transmitted from the tablet and captured. As a result, very slow or halting movements led to discontinuous recording of the measured values. For steady movements (in one stroke), the time difference between two consecutive data points was equal to the average sampling time, while it was larger for halting movements. Figure 1C,D show the time differences of the individual measurement points of the two characteristic line traces in Fig. 1A for a participant with MCI (1C, red line in Fig. 1A) and a non-MCI participant (1D, blue line in Fig. 1A). A movement was considered halting if time differences exceeded 1.5 times the sampling interval, i.e., 8.6 ms (horizontal dashed line in Fig. 1C,D). For the calculation of the ISI per trial, the difference between the actual movement duration and the number of time stamps × sampling interval (theoretical minimum movement duration) within the analysis range of the movement (y position 8–18 cm, vertical dashed lines in Fig. 1C,D) was calculated.

For further analysis, only data from subjects from whom at least four valid trials could be analyzed were used^48^. The mean value per subject and condition was calculated for all valid trials for further comparisons. Data analysis as well as statistical analysis was performed using the R 3.6.3 base package^49^. Since the data were not normally distributed, group comparisons were made using the Kruskal–Wallis test with the factors cognitive state (non-MCI and MCI) and sex (female and male). For pairwise post-hoc comparisons, Wilcoxon rank sum tests were computed. Multiple testing was performed with Bonferroni correction. Effect sizes are given as Phi coefficient (φ) and Cramer’s V (φ_c_) when appropriated. To calculate the probability of correctly classifying a participant by chance, the original classification labels were randomly redistributed 100,000 times and the proportion of correctly assigned participants was calculated for each run. The theoretical probability is given as the mean of all runs as a percentage.

To assess fine motor skills, we calculated three scores. The FM score (Table 1) is derived from the frequency of performing different tasks (answered on a 6-point Likert scale for each task, with 1 = never to 6 = very often) that require fine motor skills (playing a musical instrument, typing on a keyboard, writing, needlework, model building or other fine motor skills). The highest score for each subject was scored regardless of the task. Based on the drawing task of the CERAD-NP test battery, a drawing ability score was calculated as the sum of the 4 tests for drawing a circle, rhombus, rectangle and cube (CERAD drawing score, Table 1). Furthermore, the ratio of the duration of Trail Making Test B to Trail Making Test A was calculated (CERAD TMT B/A, Table 1).

### Ethical approval

The study was approved by the Ethics Committee of Chemnitz University of Technology, Germany, Faculty of Behavioral and Social Sciences (V232-17-KM-SENDA-07112017, approved on 19 December 2017). All methods and procedures were performed in accordance with the ethical standards of the Helsinki Declaration of the World Medical Association.

### Informed consent

Each participant signed a written in-formed consent form.

## Data Availability

The datasets analyzed during the current study are not publicly available but are available from the corresponding author on reasonable request.
